# Changes in friluftsliv (outdoor recreation) activities among Norwegian adolescents during the SARS-CoV-2 pandemic

**DOI:** 10.3389/fspor.2023.1215611

**Published:** 2023-08-11

**Authors:** Eivind Sæther, Stian Mikalsen, Pål Lagestad

**Affiliations:** Department of Teacher Education and Art, Nord University, Levanger, Norway

**Keywords:** community, physical activity, COVID-19, outdoor recreation activities, the pandemic

## Abstract

**Introduction:**

Friluftsliv (outdoor recreation) activities can provide both physical activity and experiences in nature, and improve quality of life. Regular physical activity is critical for young people's physical, social, and mental health. During the SARS-CoV-2 pandemic in 2020–2022, schools, gyms, and swimming pools were closed, and athletic teams and other physical activity services were prohibited from holding events. Despite these restrictions, access to nature and most friluftsliv persisted throughout the pandemic. The purpose of this study is to elucidate how the degree of activity in various friluftsliv activities changed during the pandemic, as well as identify the significance that friluftsliv had for the experience of community prior to and during the pandemic.

**Methods:**

To achieve this goal, 287 young people aged 16–19 answered a questionnaire.

**Results:**

The results showed that self-reported total friluftsliv decreased significantly by 12.1% during the pandemic compared to self-reported total friluftsliv activity in the year before the pandemic occurred. Modern friluftsliv experienced an overall activity decline in participation of 24.8%. Two of these activities had a significant decrease in activity level, while three of the activities had an unchanged level of activity. Traditional friluftsliv also experienced a significant decrease of 7.8%, with six of the seven friluftsliv activities exhibiting a significant decrease in activity level during the pandemic. Regarding the importance of friluftsliv for the experience of community, a substantial number of participants felt that friluftsliv activities had been more important during the pandemic than prior to the pandemic. In addition, many respondents indicated that the desire to be with friends, family, and girlfriends/boyfriends had become more important during the pandemic than before it.

**Discussion:**

The findings related to the importance of friluftsliv activities to experiencing community were not unexpected. However, it was somewhat unanticipated that the two-year pandemic did not lead to more friluftsliv among young people, given the unavailability of other avenues of physical activity—which is concerning from a public health perspective, in terms of social, psychological, and physical health.

## Introduction

From March 2020, Norway, along with most other countries globally, were largely shut down to stop the spread of the virus SARS-CoV-2 (referred to here as the pandemic). Kindergartens, schools, training centres, night clubs, and organised activities were expressly prohibited from holding events (1). At the same time, other infection control mandates and recommendations were issued, including keeping one metre apart from other people (social distancing). One was also not allowed to shake hands or hug another person, and a mask and use of antibacterial products were required (1). These restrictions limited everyone's freedom to participate in most physical and social activities. For many, friluftsliv (outdoor recreation) activities, for which there were no restrictions, constituted the only opportunity for physical activity and community. These measures were eased in Norway, as in other countries, only in February 2022. This had the effect that, for a two-year period, the population lived under strict measures that limited their opportunities for physical activity. Today's society is increasingly characterised by inactivity and sitting, and physical inactivity has, from a long-term perspective, a deleterious effect on young people's health (2). This study will examine the extent to which the pandemic changed young people's activity patterns in relation to friluftsliv activities, as well as shed light on the importance that friluftsliv has had for young people's experience of community during the pandemic.

### Friluftsliv activities

Friluftsliv, or outdoor recreation, occupies a central position in Norway, as almost the entire population practices friluftsliv (3–6), and has done so for decades (3). Friluftsliv has been defined in various ways (7–9). Norwegian authorities have defined friluftsliv as “staying outdoors and being physically active during leisure hours to have a change of environment and experience nature” (10). Friluftsliv, a literal translation of which is “free-air life”, can encompass concepts related to outdoor recreation, outdoor life, or adventure in nature (7–9, 11). However, the idea of friluftsliv comprises more than physical movement; it also indicates being inspired by and experiencing nature. Despite a declining trend in organized sports among young people (12), participation in friluftsliv seems to be stable among young people (3, 8). Research has shown that people tend to continue activities in which they participated as children (13) and, in this way, friluftsliv makes a key contribution to public health, especially among young people.

Odden (8) points out that more different social groups (according to age, gender, socioeconomic status, etc.) are participating in various friluftsliv activities compared to that in previous years. Odden (8) also emphasizes that frilufsliv is only moderately differentiated socially and geographically, even though the emergence of new activities reinforces a differentiation of friluftsliv activities. According to Fisker (14), the various trends in friluftsliv are characterised on the basis of increasing variation within friluftsliv. This diversification applies not only to new activities, but also to differentiation and specialization of the traditional friluftsliv. Fisker (14) further asserts that there is increasing individualisation in friluftsliv activities, in which friluftsliv proves to be an effective approach for experiencing activities, achieving self-affirmation, and identification through various activities. Concurrently, Tordsson (9) highlights that the difference in activity patterns between rural and urban friluftsliv is becoming less evident, signalling more similar activity trends.

In 2020, the Norwegian Environment Agency, the Norwegian Directorate of Health, and various friluftsliv organizations presented nine common guidelines for good and safe trips during the pandemic (15). These guidelines were called “Corona rules for friluftsliv”, and specified how Norwegians could engage in friluftsliv in local areas in a safe manner with their own social groups. Here, it was argued that Norwegians have rarely had a greater need to be physically active in the fresh air and, simultaneously, have so many available friluftsliv activities to engage in during the pandemic.

### Friluftsliv as a socio-cultural phenomenon

In addition to the fact that friluftsliv activities can positively affect physical and mental health, they also constitute an arena for developing social and interpersonal skills (4, 9). Both Tordsson (9) and Mytting and Bischoff (4) report that participation in friluftsliv activities is critical for developing responsibility, caring, and empathy. Moreover, Tordsson (9) and Gurholt (11) highlight participation in friluftsliv as a vital socio-cultural phenomenon—a phenomenon that arises and develops through historical, social, and cultural conditions. In this way, engagement with nature through friluftsliv provides the space and opportunity for identity development for everyone, and especially for young people (16). During adolescence, the individual's identity is created through intense emotional activity (16), much of it involving interaction with peers. Skårderud (16) also states that meetings between people can be similar to what occurs on a stock exchange, “It is exchanged and traded. The transactions are not financial, but psychological and social. It is, and is about, social recognition and self-esteem” (16). Identity is, thus, not predetermined, but instead something that the individual must find and create in cooperation with others (9). Adolescence can be characterised as a time replete with many and strong emotions, puberty, separation from parents, and a greater need for socialising with peers (11, 17).

Broch (17) asserts that one of the worst consequences of the pandemic in terms of young people was the loss of contact with peers. During large parts of the pandemic, social meeting places were closed. Moreover, at the start of the pandemic, contact was restricted only to those in one's household group. As a consequence, many had much less contact with friends, relatives, and girlfriends/boyfriends during the pandemic compared to before the pandemic. From a biological perspective, humans can be considered to be an ultrasocial herd animal and, thus, not being able to meet with family and friends can put people in a state of crisis. At the same time, it should also be noted that humans are remarkably adaptable. Tjora (18) views community as a social reality in which participants will have different experiences of collective consciousness. Collective consciousness is defined by Tjora to include having the same outlook on life, corresponding ideas, and moral attitudes. Indeed, humans have a definite perception of cohesion and agreement, and notice whether or not there a community is present. Tjora further emphasises that people have a feeling that they are part of a community, not just as an individual experience, but one that is closely linked to a tangible reality. The concepts of interdependence, solidarity and interaction are also central, and refer to distinct aspects of community: to belong together; to stay together; and to do something together. Tjora (18) emphasizes the importance of interdependence in identifying with one another, such as with others who engage in friluftsliv. Solidarity refers to standing together in solidarity, especially during and after a crisis, such as the pandemic. Interaction entails being together and doing something together, e.g., an outdoors activity. While Tjora (18) approaches community from the outside, Tugetam (19) and Broch (17) attempt to understand youth, community, and outdoor life from the inside by listening to young people's own voices. The expression “to be in the same boat” speaks to the mutual dependence and community represented by friluftsliv (20). In this context, friluftsliv can function as an essential meeting place for social experiences and community. The associations “Medvandrerne” (21) and “#sammenpåtur” (22) are examples of organisations that promote both friluftsliv and community experiences. In the Year of Volunteering 2022, the organisation Norsk friluftsliv (The Norwegian Association for Outdoor Organisations) designated “touring in community” as a central focus to stimulate outdoor recreation, promote togetherness, and prevent loneliness.

### Relatedness as a psychological need

In relation to friluftsliv activities as an arena for social relationships, we use Deci and Ryan (23) self-determination theory as an important theoretical lens. Self-determination theory emphasizes the importance of students’ social environment for personal growth. Deci and Ryan (23) argue that three psychological basic needs exist in all humans: autonomy; relatedness; and competence. These factors are essential for optimal motivation, integration, wellness and well-being which, in turn, lead to intrinsic motivation. Intrinsic motivation refers to performing an activity because it is inherently interesting and provides its own reward by satisfying one's basic needs for autonomy, competence, and/or relatedness (24). Internal motivation comes from within, and leads to the kinds of behaviors that one wants to perform. External motivation, on the other hand, derives from an external source. Internal motivation is especially essential for children and young people in terms of a lifelong delight in movement; it is also closely connected to learning, and is the prototype of self-determination in self-determination theory. Self-determination theory emphasises the significance of the social environment of students for personal growth.

In the present study, relatedness is also of great importance. Relatedness refers to feeling connected to others, to caring for and being cared for by those others, and to having a sense of belongingness both with other individuals and with one's community (25). Deci (26) claimed that adolescents need to feel connected with others, i.e., to care and be cared for (i.e., the need for relatedness). Furthermore, Deci (26) maintained that human behaviour and experience should be viewed in terms of the dialectic between the person and the environment. The interaction between the active organism striving for autonomy and the social context can be either nurturing or antagonistic toward a person's tendencies. Although self-determination theory constitutes a theoretical perspective in this study, it is worth mentioning that this study will not examine relatedness in friluftsliv activities. Relatedness, however, is an important psychological need for young people—a need that could be fulfilled with friluftsliv activities during the pandemic because these activities were not shut down during this period. With such a strategy, relatedness (as a basic psychological need) is an important framework for the study.

### Previous research on relatedness

Several empirical studies underscore the relevance of self-determination theory, and its importance related to well-being and social belonging, as well as its association with increased physical activity level (27–31). For example, Oldervik and Lagestad (30) found that self-determination positively affected physical activity level among adolescents, and their results indicated that increased self-determination positively affected their happiness and well-being.

### Previous research on friluftsliv activities in Nordic countries during the pandemic

Previous research has revealed that many young people took advantage of friluftsliv activities during the pandemic. According to a Statistics Norway's survey from 2021 of living conditions for Norwegians between the ages of 16 and 24 (6), a total of 98.8% of the respondents had participated in friluftsliv. Specifically, 88.1% had gone on a hike, 25.1% had gone on a berry- or mushroom-picking trip, 27.3% had gone cycling, 38.4% went alpine skiing, snowboarding, or cross-country skiing, 45.2% had fished, 8.8% had hunted, and 12.1% engaged in outdoor climbing (6). In fact, data from Norsk Friluftsliv (32) on outdoor participation during the pandemic showed that 32% of Norwegians had engaged in friluftsliv activities more often during the pandemic than before the pandemic. The same survey reported that those under the age of 40 increased their activity level the most; whereas, young people between the ages of 15 and 24 stated that they engaged in less friluftsliv (24%). Nature walks was the most popular activity that increased during the pandemic, constituting an increase of 39%. Moreover, 65% used outdoor activities with the aim to be physically active and get exercise during the pandemic. Swedish research has also indicated that many young people took advantage of friluftsliv during the pandemic. In a Swedish survey among students, 64% of 442 respondents stated that they had changed their friluftsliv habits during the pandemic (33). 62% of the students stated that they made use of nature more often than prior to the pandemic. 56% responded that they spent more time with friends and family in nature during the pandemic. 11%, however, replied that they participated in friluftsliv less often during the same period. The most popular friluftsliv activity for these students was walking (79%), while other activities, such as cycling (21%), mushroom- and berry-picking (17%) and ski trips (17%), had less support. The students enjoyed friluftsliv activities with their friends (27%), girlfriends/boyfriends (27%), and families (25%). 20% of the students enjoyed friluftsliv alone. In another Swedish survey, it was reported that many students changed their outdoor habits during the pandemic (34). 51% had increased their friluftsliv activity during the pandemic. The same study also indicated that people engaged in friluftsliv activities more often for physical activity, such as walks, runs and bike rides, during the pandemic, and that nature became a vital social arena for them.

### Research questions

The discussion above has pointed to friluftsliv as an important arena for young people's physical and social activity during the pandemic. While numerous studies have been carried out in this field, there is a paucity of research that examines changes in friluftsliv activity before and during the pandemic among the same respondents. With such an approach, the two research questions are as follows: (1) How has the pandemic changed young people's level of activity in friluftsliv activities, in general, and within traditional and modern friluftsliv activities; and (2) To what extent has the meaning of participation in friluftsliv activities changed during the pandemic, in terms of young people's experience of community?

## Method

To illuminate these issues, responses were collected using a questionnaire. The study was approved by the Norwegian Centre for Research Data (reference number 261981). In accordance with Norwegian regulations, participants at the age of 16 or older have to provide written consent to participate in the study before data collection can begin, and this regulation was followed.

### Participants

The sample in this research project was drawn from students at two upper secondary schools in central Norway located in medium-sized cities (between 10,000 and 20,000 inhabitants). These two schools were selected using a stratified sample. Both schools are in areas with abundant countryside, access to several national parks, rivers, the sea, mountains, ski slopes, and alpine resorts. Due to time constraints, the principals and teachers would not allow every class to participate, and thus the principals randomly selected classes to answer the questionnaire. Out of the 900 pupils at school 1, classes with 140 pupils were selected, while classes with 147 out of 1,250 pupils were selected at school 2, and all of these completed the questionnaire (*N* = 287). The pupils in these schools ranged in age between 16 and 19, corresponding to three years of upper-secondary school. Classes from all three years were selected. 135 boys and 152 girls answered the questionnaire, which were divided into sports subjects (*N* = 114), specialisation in general subjects (*N* = 103), and vocational subjects (*N* = 70). Because of the random selection of the classes and the distribution of age, main subjects and gender, we assert that this sample is representative of Norwegian youth.

### Implementation

Based on an extensive literature search, a questionnaire was created that asked respondents about their participation in friluftsliv activities before and during the pandemic. The questionnaire consisted of five questions about the frequency of five modern friluftsliv activities, and seven questions about the frequency of seven traditional friluftsliv activities, both before and during the pandemic. These 12 friluftsliv activities are defined and well-known among Norwegians. In these 24 questions, the participants reported the number of trips/times that they had engaged in each activity during the last year before the pandemic (from March 2018 to March 2019), and the last year during the pandemic (from February 2021 to February 2019). There were also seven questions that used a five-point Likert scale that sought to illuminate the young people's experience of the importance of friluftsliv activities for their sense of community during the pandemic compared to before the pandemic (“much less important”, “less important”, “equally important”, “more important”, and “much more important”). Finally, at the end of the questionnaire, the participants were asked to answer the following open-ended question: “Describe what was the most positive aspect of your use of friluftsliv activities during the pandemic.” A pilot study was completed among four high school students from another high school, and some questions were rewritten based on feedback from these students, so that acceptable validity and reliability were obtained. This feedback was related to unclear formulations of the questions. The data collection occurred in February 2022, took place in the classroom setting with the teacher present, and took 15 min. This strategy likely explains why all of the students completed the questionnaire.

### Analyses

To address the research questions, descriptive statistics (percentage) and statistical analyses were used. The assumption of normality according to two dependent variables (activity level and social relationship) was not met (*p* < 0.05) in a Kolmogorov-Smirnov test (26), and non-parametric tests had to be used. To examine differences between activity level before and during the pandemic, Wilcoxon Signed Ranks non-parametric tests were employed (35). To determine the importance of social relationships before and during the pandemic, descriptive data (mean and standard deviation) were selected. The significance level was set to *p* < 0.05. All statistical analyses were carried out in SPSS, Version 25 (IBM, Armonk, NY, U.S.A.). After the statistical analyses were performed, the qualitative data from the open-ended question were transcribed and analysed with the aim to achieve meaning condensation, in accordance with Kvale and Brinkmann (36). This analysis process was intended to explore categories that could explain the main findings from the statistical analyses. Using interpretation during a process of reading through the participants’ reflections several times, categories were formed related to both research questions: friluftsliv activity and community. In this way, the meaning of the text data was condensed, by making categories related to the meaning of the participants statements (36), and several categories were established. According to the research question about change in activity level, two categories were created by analysing the data: “nothing” and “more positive comments towards traditional friluftsliv activities”. For the research question about how the meaning of participation in friluftsliv activities changed during the pandemic, in terms of young people's experience of community, two categories were created: “more relationships” and “better relationships”.

## Results

### Friluftsliv activities before and during the pandemic

**Figure 1:**
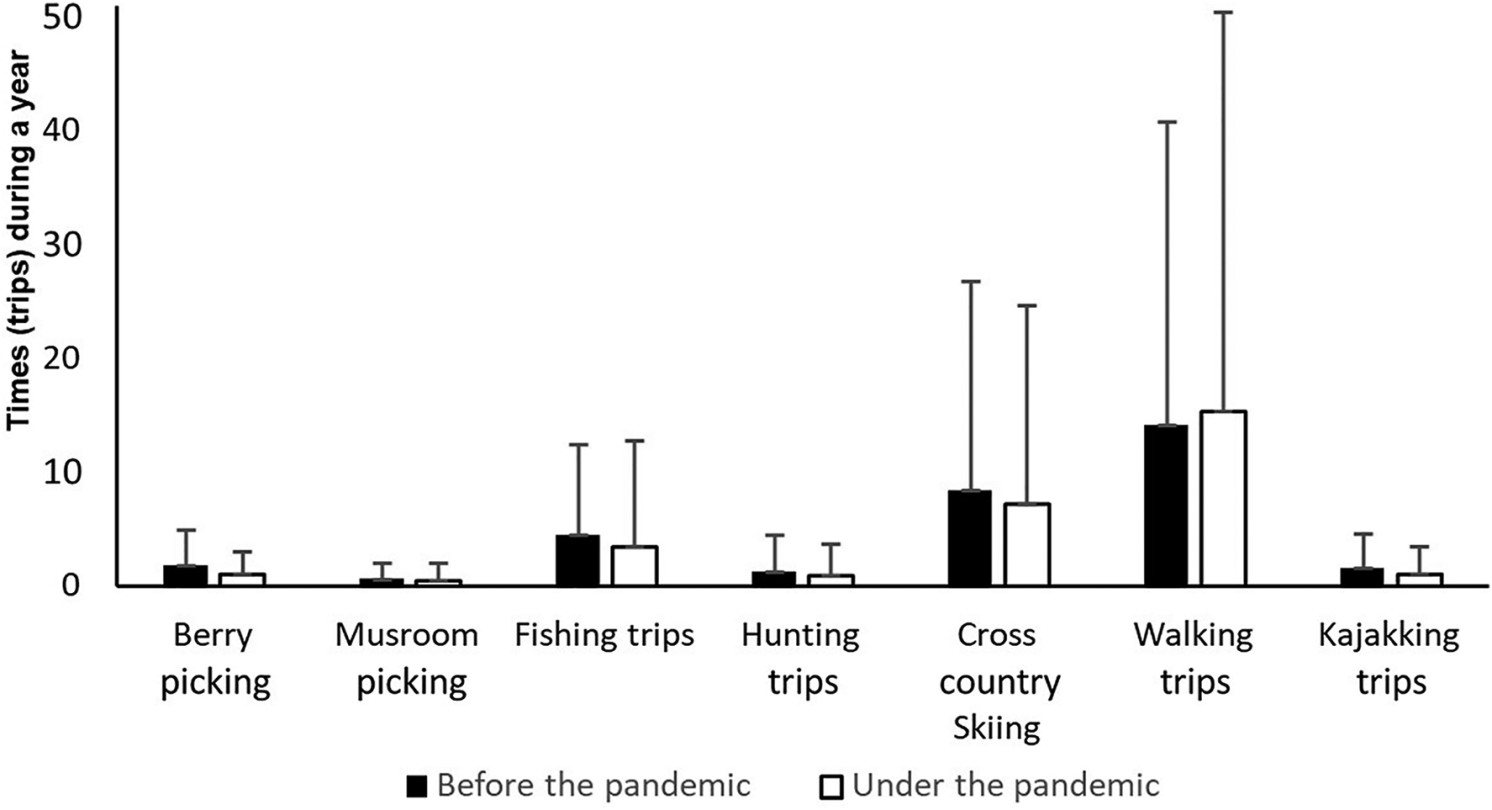
Number of times that the respondents performed traditional friluftsliv activities in the year before the pandemic and in the last year during the pandemic.

**Figure 2:**
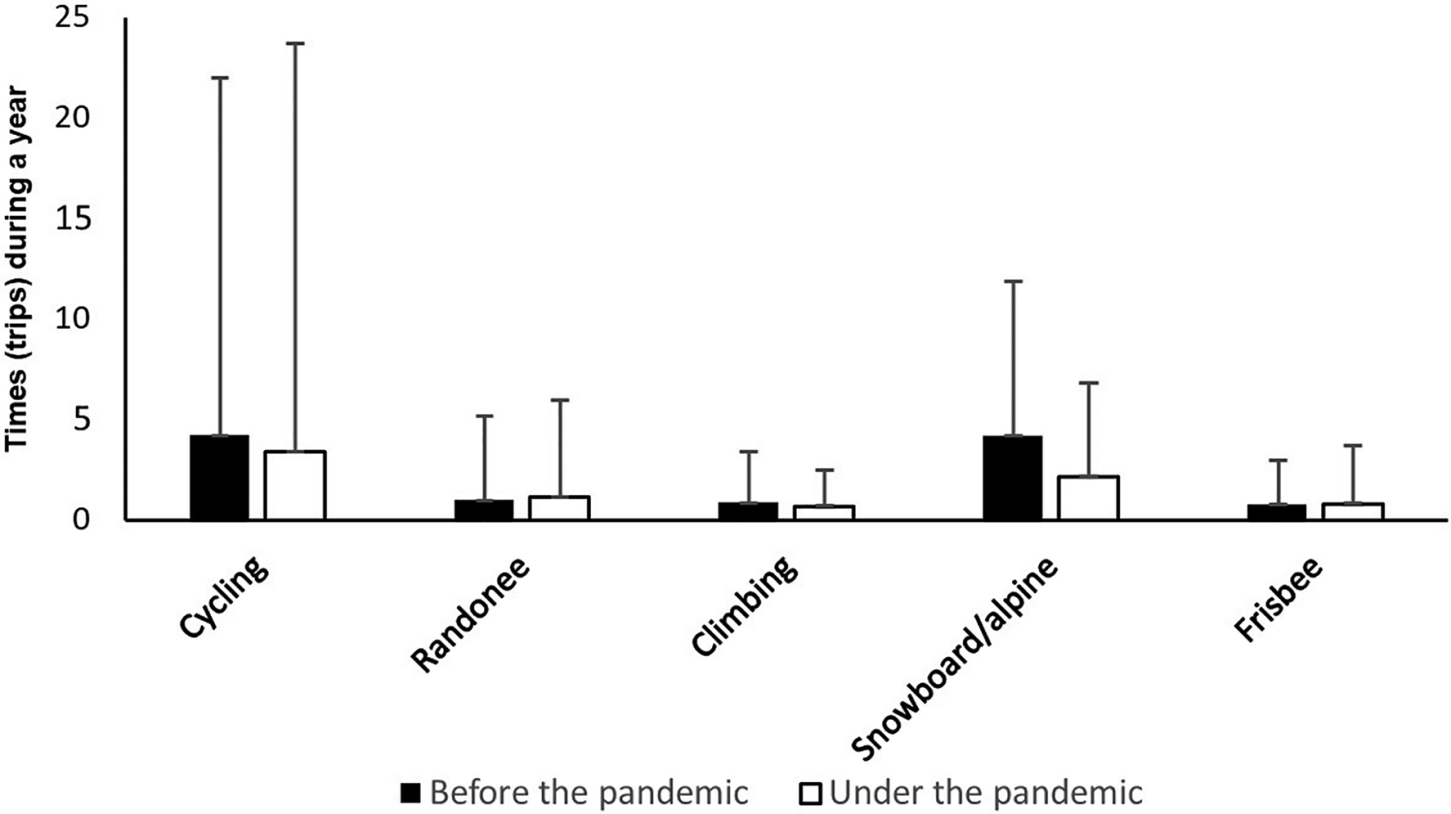
Number of times that the respondents performed modern friluftsliv activities in the year before the pandemic and the last year during the pandemic.

The respondents reported performing the various friluftsliv activities, on average, 43.4 times (SD = 53) in the last year before the pandemic, while the average number during the pandemic was 38.1 (SD = 62.3). This constitutes a significant decrease of 12.1% (*Z* = −5.82, *p* < 0.001). The change in the number of times that the respondents engaged in traditional friluftsliv activities and modern friluftsliv activities in the last year before the pandemic, in the last year during the pandemic, can be seen in Figures 1, 2, respectively.

Statistical analyses reveal that traditional friluftslliv activities overall exhibited a significant decrease of 7.8% from the year before the pandemic to the last year during the pandemic (*Z* = −4.8, *p* < 0.001). However, variations exist in how much the activity decreased in the various traditional friluftsliv activities. Further analysis indicates a significant decrease in six out of seven traditional friluftsliv activities in the degree of activity during the pandemic. This applies to berry- and mushroom-picking trips, fishing trips, hunting trips, ski trips, and kayaking/canoeing (*Z* = −7.1, *p* < 0.001; *Z* = −3.3, *p* < 0.001; *Z* = −6.8, *p* < 0.001; *Z* = −2.6, *p* < 0.01; *Z* = −4.2, *p* < 0.001; *Z* = −3.3, *p* < 0.01). The activity of walking in forests and mountains showed an insignificant increase of 8.9% in the last year during the pandemic compared to the year before it (*Z* = −0.6, *p* = 0.577).

Statistical analyses showed that modern friluftsliv activities overall exhibited a significant decrease of 7.8% from the year before the pandemic to the last year during the pandemic (*Z* = −4.8, *p* < 0.001). Further analysis revealed, however, that three of the modern friluftsliv activities had an unchanged level of activity (*p* > 0.05), which are running, frisbee golf, and climbing. The modern friluftsliv activities, mountain biking and snowboarding/alpine skiing, showed a significant decrease in activity levels from the year before the pandemic to the last year during the pandemic, of 19% and 47.7%, respectively. (*Z* = −4.5, *p* < 0.001; Z = −10, *p* < 0.001).

### Community-related friluftsliv activity before and during the pandemic

**Figure 3:**
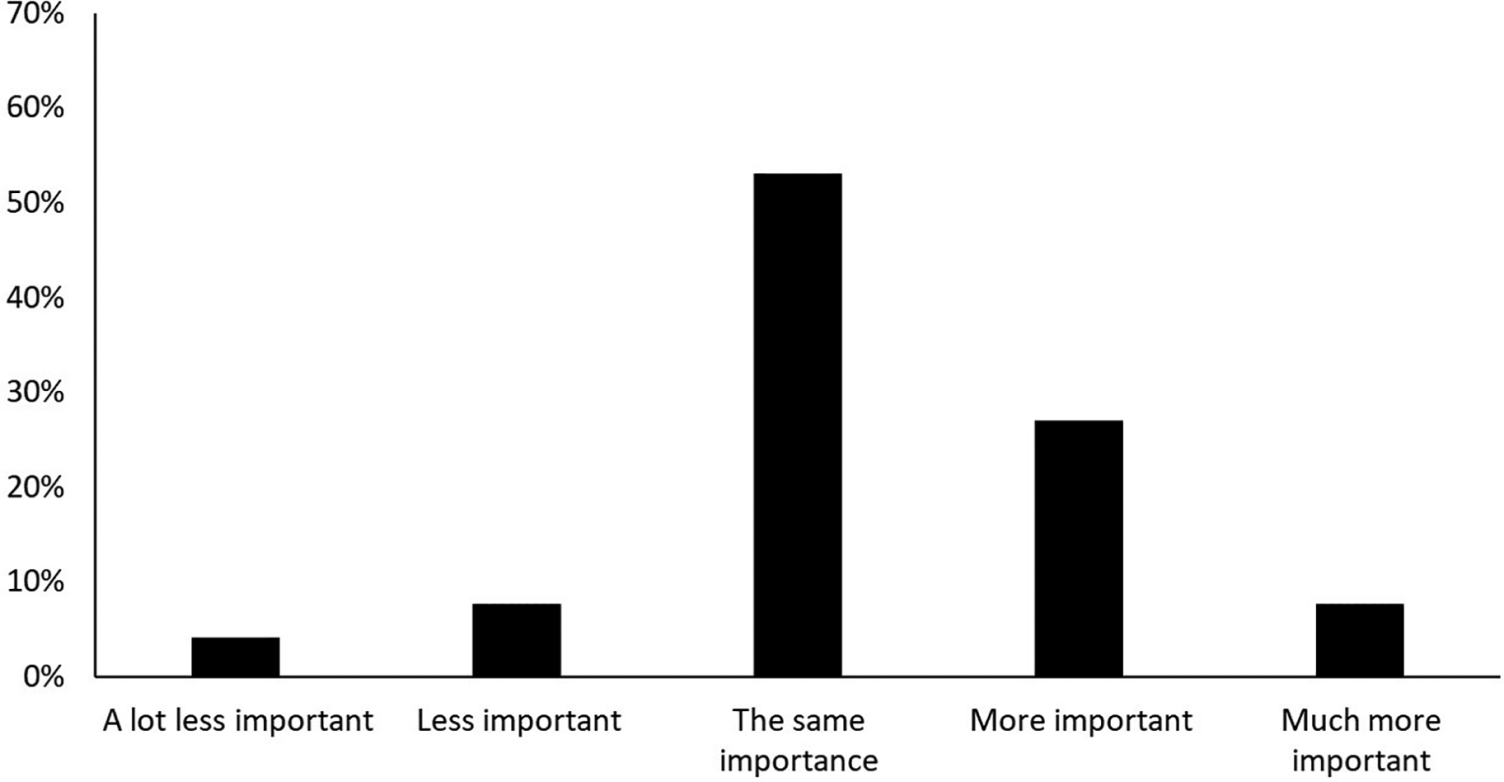
How important friluftsliv activities are for the feeling of being part of a community before and during the pandemic.

The second part of the research assesses the importance friluftsliv activities in fostering a sense of community before and during the pandemic. Figure 3 shows how important friluftsliv activities are for the feeling of being part of a community before and during the pandemic.

Figure 3 shows that 11.9% of respondents’ answered that friluftsliv activities were less or much less important to them for the sense of being part of a community during the pandemic compared to before the pandemic. However, a markedly larger 34.8% responded that friluftsliv activities became more important or much more important regarding the feeling of being part of a community during the pandemic compared to before it.

**Figure 4:**
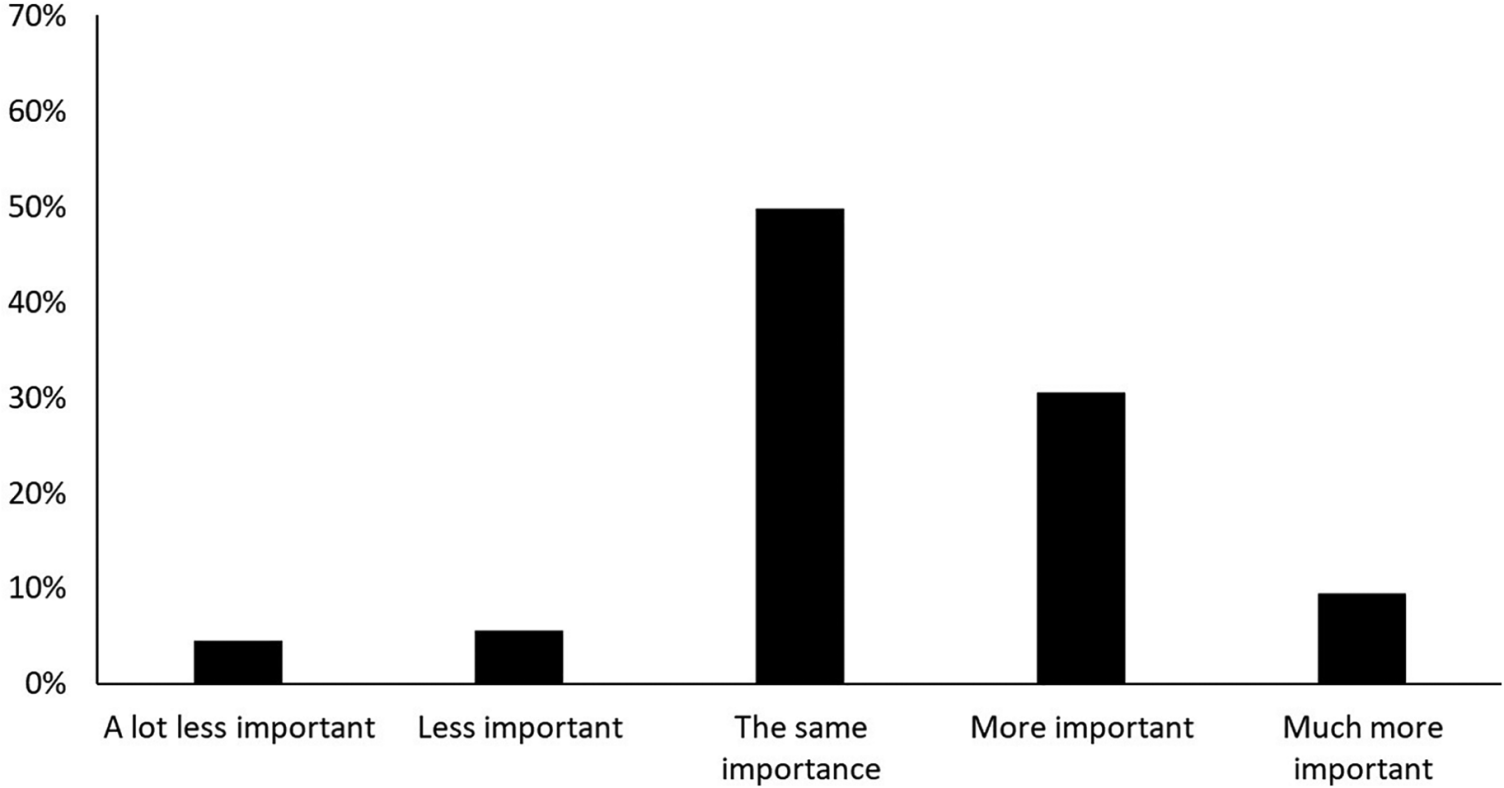
How important friluftsliv activities were in being able to be with friends during the pandemic and before the pandemic.

Figure 4 shows how important friluftsliv activities were in being able to be with friends during the pandemic and before the pandemic.

As illustrated in Figure 4, 10.2% of the students responded that friluftsliv activities were less or much less important for them to be able to be with friends during the pandemic compared to before the pandemic; whereas, 40% responded that outdoor activities were more important or much more important for them in being able to be with friends during the pandemic compared to before the pandemic.

**Figure 5:**
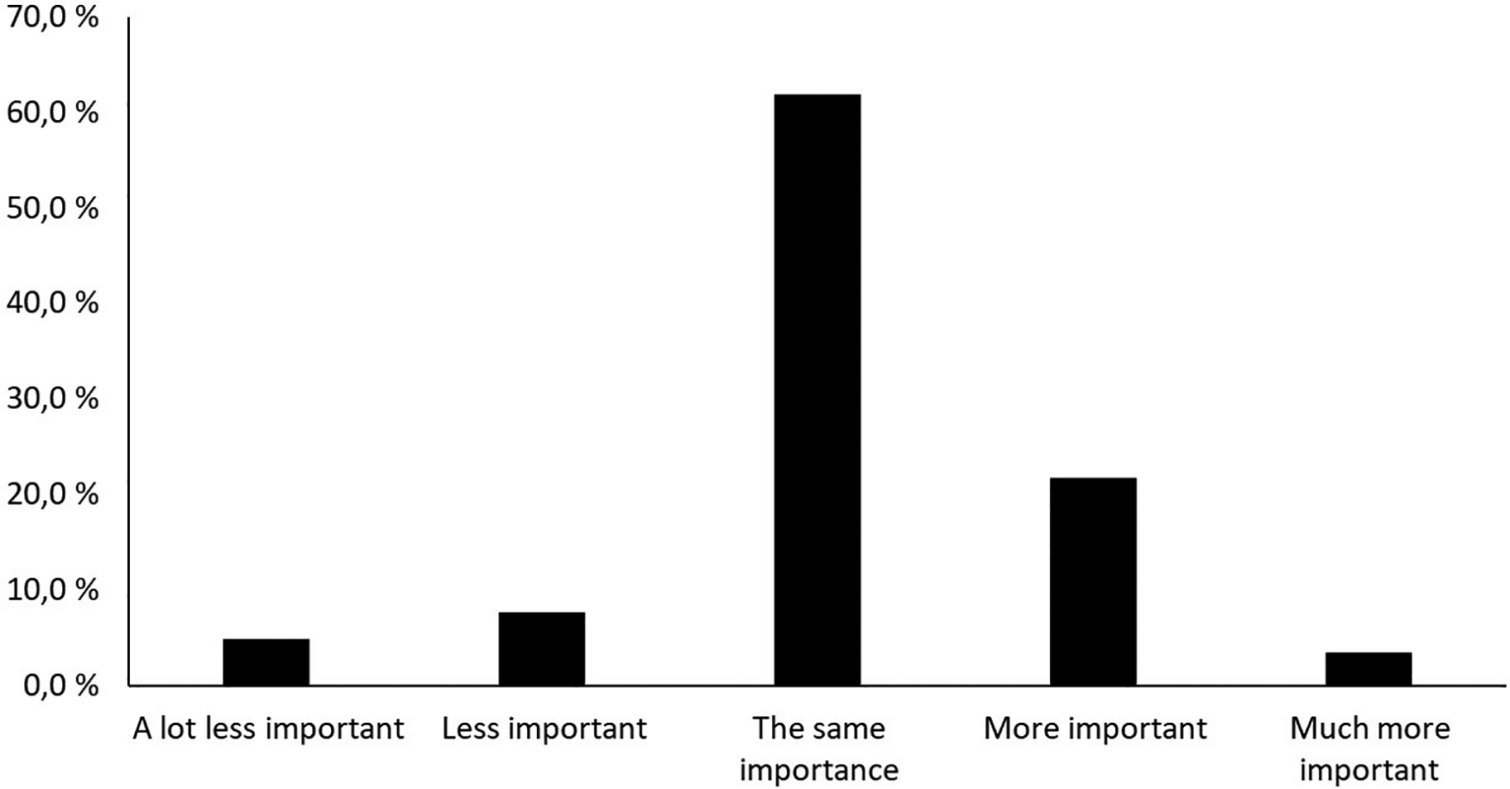
How important friluftsliv activities were in being able to be with family members during the pandemic and before the pandemic.

Figure 5 shows how important friluftsliv activities were in being able to be with family members during the pandemic and before the pandemic.

Figure 5 shows that, while 12.6% responded that friluftsliv activities were less or much less important for them to be able to be with family members during the pandemic compared to before the pandemic, 25.3% responded that friluftsliv activities were more important or much more important for them in being able to be with friends during the pandemic compared to before it.

**Figure 6:**
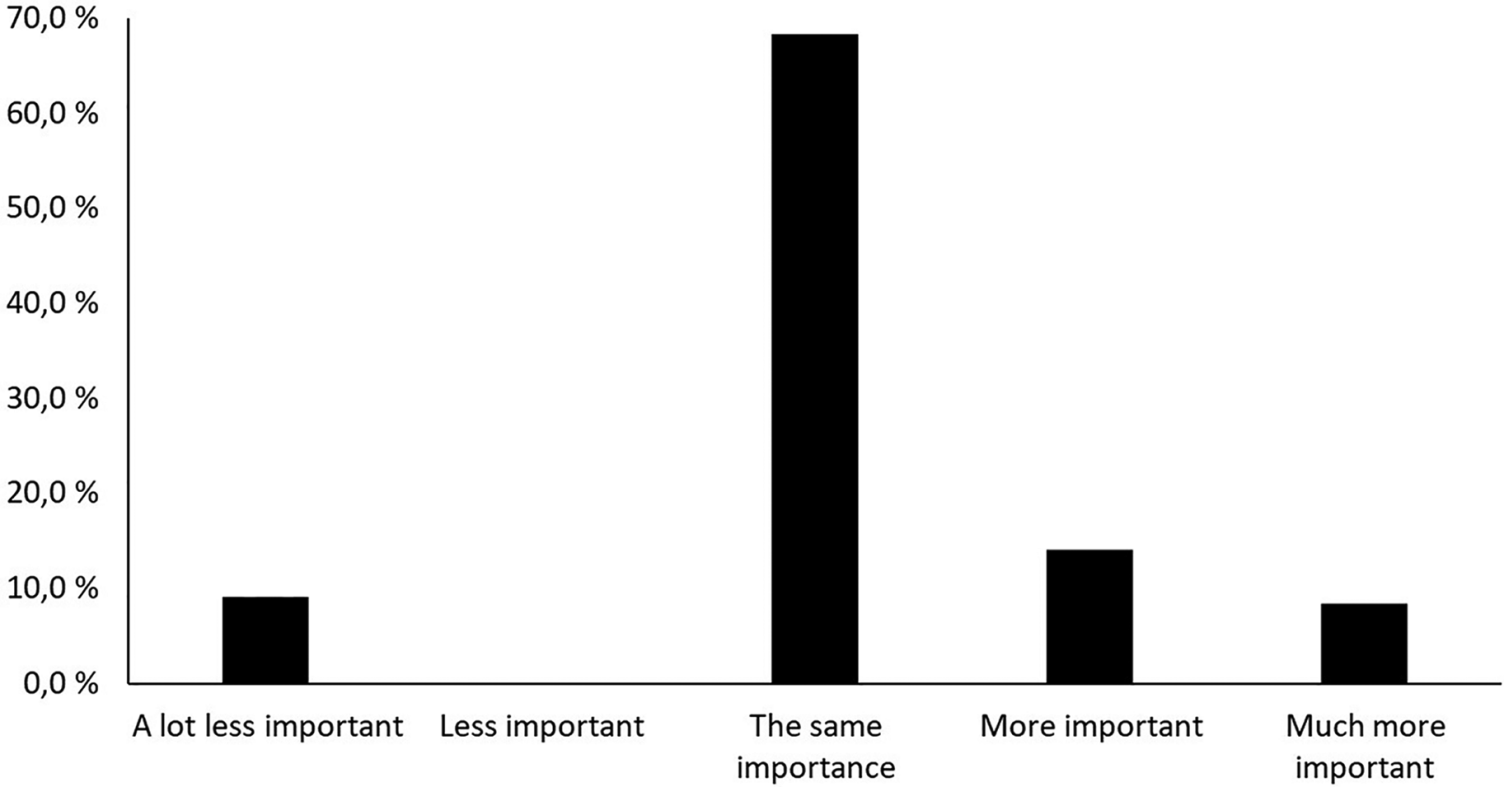
How important friluftsliv activities were in being able to be with a girlfriend/boyfriend during the pandemic and before the pandemic.

Figure 6 shows how important friluftsliv activities were in being able to be with a girlfriend/boyfriend during the pandemic and before the pandemic.

Figure 6 shows that, while 9.2% of the students responded that outdoor activities were less or much less important for them to be able to be with a girlfriend/boyfriend during the pandemic compared to before it, 22.6% responded that friluftsliv activities were more important or much more important for them in being able to be with a girlfriend/boyfriend during the pandemic compared to before the pandemic.

### Qualitative results

Qualitative analysis of the open-ended question showed that 51 of the participants answered “nothing” to the question about what was the most positive aspect of the friluftsliv activities during the pandemic. However, while over 80% of the participants had a positive answer to the open-ended question, substantially more positive comments were received regarding traditional friluftsliv activities compared to modern friluftsliv activities. In terms of traditional friluftsliv activities, the participants indicated that hunting trips were the most positive aspect because the school was locked down. Many also identified “just go out and walk”, “peace”, “silence”, “fresh air”, and “freedom” when they stated the most positive aspects of friluftsliv. However, positive remarks related to modern friluftsliv activities were fewer, but words, such as “up on the top”, action”, “speed”, and “excitement”, were mentioned.

As previously stated, concerning the research question about how the meaning of participation in friluftsliv activities changed during the pandemic in terms of young people's experience of community, the qualitative analyses revealed two categories: “more relationships” and “better relationships”. “More relationships” is the concept signifying the critical importance of maintaining social relationships through the act of engaging in friluftsliv activities. Many of the respondents directly identified social relationships as the most positive aspect of participating in friluftsliv during the pandemic. The reflections of the participants indicated that social relationships are a very important outcome of engaging in friluftsliv activities. In fact, several participants viewed friluftsliv activities as the only opportunity for social relationships during the pandemic, citing reasons, such as “to still be with friends”, “unity”, and “the possibility to meet other people”, as the most positive aspects of engaging in friluftsliv during the pandemic. “Better relationships” concerns the position that friluftsliv activities hold in the lives of the participants, as they are viewed as enhancing the quality of social relationships. Participants identify friluftsliv as the “best strategy for relationships” and to “increase the quality of relationships”. “Quality time with my Dad”, “bonfire nights with the boys”, and “more connected to friends” are statements associated with the most positive aspects of engaging in friluftsliv.

## Discussion

### Friluftsliv activities before and during the pandemic

#### A decrease in friluftsliv activities during the pandemic

The first main finding was that the activity level of friluftsliv activities was significantly reduced during the pandemic, by 12%. This result was somehow unexpected, given that we assumed that the activity level of friluftsliv activities would increase during the pandemic. However, extant literature focusing on Norwegian youth supports our findings. For example, Norsk friluftsliv (32) reported that, while Norwegians over the age of 24 engaged in more friluftsliv activities during the pandemic, 24% of Norwegians between the ages of 15 and 24 reported engaging in less friluftsliv activities during the pandemic compared to before it. The fact that multiple studies indicate a decrease in friluftsliv activity among young people during the pandemic is concerning from a health perspective, with respect both to physical and mental well-being. This is because sports teams and gyms closed down for large parts of the pandemic. Physical activity during youth was also demonstrated to be positively associated with several major health benefits, such as positive changes in skeletal health, cardio-respiratory fitness, and adiposity, as well as quality of life (37, 38). This reduction took place even though the Norwegian Environment Agency released guidance on how friluftsliv activities could be performed during the pandemic (15).

While our survey among young Norwegians finds a reduction in friluftsliv activity during the pandemic of 12.1%, Swedish surveys, in contrast, show an increase among young Swedes. Among Swedish students, Kindal and Wiklund (33) observed an increase in friluftsliv activity during the pandemic of 62%. Furthermore, a study by Hansen et al. (34) found that Swedish young people increased their friluftsliv activity by 51.1%. Even though the Swedes engaged to a greater extent in more friluftsliv activities during the pandemic compared to before the pandemic, the literature indicates that Norwegian youth nevertheless performed more friluftsliv activities than Swedish youth during the pandemic. This is because research indicates that almost all Norwegian young people engage in friluftslliv activities, friluftsliv activities are significantly more ubiquitous among Norwegians than Swedes (3–5, 33, 34), and friluftsliv activities have a unique position in Norway (8). In this respect, we argue that the potential for an increase in friluftsliv activity is greater in Sweden, as there are more people who did not engage in friluftsliv activities before the pandemic compared to Norway, and the conditions for friluftsliv activities are equally facilitative in both countries. We also emphasise that, even though our data from 2022 show a decline in young people's friluftsliv activities during the pandemic compared to Statistics Norway's data (6), the young people in our study from 2022 performed friluftsliv activities more often than Rafoss and Seippel (5) found in their six-year-old study.

#### A larger decrease in modern friluftsliv activities than traditional friluftsliv activities

The second main finding was that there was a significant reduction in activity level during the pandemic in both traditional and modern friluftsliv recreation activities. However, while the reduction was 7.8% in traditional friluftsliv activities, it was much greater for modern friluftsliv activities, constituting a decrease of 24.8%. This can be explained by the fact that traditional friluftsliv is simple, easily accessible, and inexpensive (4); whereas, modern friluftsliv necessitates risk, expensive equipment, and expertise (8), as well as more planning. The finding may also be related to the fact that young people have not had the opportunity to practice traditional friluftsliv activities through school. Although some young people in our study expressed that they had more time in their everyday lives, the results show that they have not used this time to engage in more friluftsliv during the pandemic. The analysis of the open-ended question also revealed that many of the participants answered “nothing” on the question related of what was the most positive aspect of friluftsliv activities during the pandemic. On the other hand, our findings may be explained by a more positive attitude towards traditional friluftsliv activities—a finding revealed from the qualitative analyses. Overall, substantially more positive comments were given about traditional friluftsliv activities compared to modern friluftsliv activities.

Furthermore, we assert that traditional friluftsliv activities were more available than modern friluftsliv activities, i.e., some of the modern friluftsliv activities were shut down during the pandemic. During the winter, alpine resorts were closed, with the result that the rental of alpine ski equipment, including cross-country skiing equipment, was not available. In the summer, rental options for important outdoor equipment, such as canoes and kayaks, were also non-existent. It was, thus, only the simplest forms of friluftsliv that were available, which was also documented by other investigations (6). Moreover, new and modern activities are, to a greater extent than traditional activities, practiced by young people. Furthermore, the young people in our study were 16–19 years old. Statistics Norway's data comprise young people in the 16–24 age range, i.e., somewhat older young people. Young people between 20 and 24 actually belong to a different group of young people who have often started their adult life by studying or working and moving away from home, resulting in their having their own leisure preferences. It is appropriate here to acknowledge the markedly high standard deviations in several of the activities, which were sometimes three times higher than the mean. These high numbers show that there large variations exist between the adolescents regarding their activity level in friluftsliv. The fact that our findings concerning friluftsliv activity are consistent with larger Norwegian population surveys strengthens the validity and reliability of our findings.

#### The development of activity level in individual friluftsliv activities

The most prominent findings within the individual friluftsliv activities will be now discussed. Our research corroborates Norwegian (32) and Swedish surveys (33) that identified walking as the most common activity during the pandemic. Going for a walk is a simple form of friluftsliv (4), and it is still the activity with the greatest support. Many tourist cabins in attractive areas and in the mountains were closed during the pandemic, which may have had an impact on where young people went on walks. Overall, shorter trips require less effort (8). Walking is also the easiest activity to carry out, as it requires neither special equipment nor domain-specific knowledge, and can be performed directly from home and is relatively independent of weather conditions. Our results indicate that there was a large decrease in the share of the “harvesting activities” of berry trips during the pandemic compared to before the pandemic. Moreover, the decrease in the number of times that the activities were performed in the last year during the pandemic was huge for berry trips and fishing, which was an unexpected result. This decline can be ascribed to the fact that some young people were often on berry and fishing trips prior to the pandemic, and that these had a major impact on the overall decline in activity, since they were less likely to go on berry and fishing trips during this time. This may also particularly apply to berry tours since this activity had an especially low turnout. Mushroom-picking and hunting are other harvesting activities that had a decrease in activity. Mushroom trips and hunting are activities that are less widespread, and are often performed by people with special interests. Four students stated that harvesting was the most important activity for them in friluftsliv during the pandemic. Here, too, the decrease in the number of times can be explained by the fact that some students who performed these activities frequently simply limited the activity, or did not do the activity at all, during the pandemic.

Skiing is another activity in which a decline was identified. In essence, fewer people engaged in skiing during the pandemic, which assists to account for the decrease in the overall frequency of this activity. During the pandemic, schools were either closed or partially closed. Because of the closed schools, the traditional skiing day and other friluftsliv days/trips, which are popular in schools, were discontinued. This may explain the decline, as some students only participate in skiing activities when they are school-led.

During the pandemic, snowboarding and alpine skiing decreased in popularity compared to before the pandemic. The reason for the decline may be that the alpine resorts were closed in Norway during extended periods of the pandemic. There were also certain rules, such as restrictions on group sizes, social distancing measures, and the cancellation of ski races, which further reduced activities at the alpine resorts (39). In addition, the border to Sweden was closed. For many of those who live in this area, it is normal to take trips to Sweden to go snowboarding and/or alpine skiing. In fact, a large part of the population has a cabin in Sweden, contributing to its popularity as a destination for these activities. This is especially true during Easter, the winter holidays, and on weekends.

### Community-related friluftsliv activity before and during the pandemic

The third main finding revealed that, even if half of the respondents stated that engaging in friluftsliv was as important before the pandemic as during the pandemic in terms of social relationships, more respondents pointed to friluftsliv as significantly more important during the pandemic (34.8%). This was also the case in terms of social relationships with friends, family, and girlfriends/boyfriends, in which more respondents identified friluftsliv as significantly more important during the pandemic. This result was supported by the finding of the qualitative analyses, indicating “more relationships” and “better relationships” in terms of frilutsliv activities during the pandemic. Many of respondents directly identified social relationships as the most positive aspect of engaging in friluftsliv during the pandemic. Several participants emphasized that friluftsliv activities were the only arena for social relationships during the pandemic, and to still be with friends and have possibility to meet other people as the most positive aspects of engaging in friluftsliv during the pandemic. Moreover, the quality of relationships during friluftliv activities were highlighted as important by the participants.

The findings can be explained by the previously introduced self-determination theory of Deci and Ryan (25), in which relatedness is one of three basic psychological needs in all humans. Relatedness entails feeling connected to others, caring for and being cared for by those others, fostering a sense of belongingness both with other individuals and with one’s community. Relatedness is essential for optimal motivation, integration, wellness, and well-being among adolescents. We assert that our findings indicate that the need for relatedness could be fulfilled with friluftsliv activities during the pandemic because these activities were not shut down during the pandemic. With such a theoretical framework, our study underscores the importance of friluftsliv activities as a venue for social belonging.

#### Friluftsliv activities as an arena for social belonging

Our study showed that 11.7% of respondents consistently engaged in friluftsliv activities with others, 2.8% preferred to do friluftsliv activities alone, while the remaining 85.5% participated in friluftsliv activities both alone and with others. All of these findings depict friluftsliv not only as a significant space for engaging in physical activity, but also for being social with others. This argument is supported by the fact that 88% of the respondents reported that friluftsliv was of equal or greater importance in terms of community involvement during the pandemic. Further studies also indicate that friluftsliv is crucial in terms of social relationships, even if this was not examined directly. According to Hansen et al. (34), the countryside was perceived as an important social setting for young Swedes. Kindal and Wiklund (33) found that 80% of Swedish students engaged in friluftsliv activities with others, while 20% practiced friluftsliv activities alone. It is worth noting here that several young people wrote in open responses in the questionnaire that meeting others outdoors was most important to them during the pandemic. We posit that friluftsliv provided a social platform for young people that disappeared when the pandemic occurred, and many social meeting places (e.g., fitness centres) were closed. The findings from our study indicate that, during the pandemic, young people experienced friluftsliv as particularly important from a social perspective, since other social arenas, where they could meet friends, such as school, sports clubs, and gyms, were closed or partially closed. In particular, the walking activity is well suited to providing the opportunity to be with friends, family, and girlfriends/boyfriends during the pandemic. The mere act of getting out in the fresh air or engaging in movement with others may have resulted in the increase that we found in the activity of walking.

Our findings indicate that the concept of the collective unconscious, as described by Tjora (18), resonated with young people during the pandemic when they were banned from many social spaces, thus leading them to value friluftsliv more for fostering a sense of a community. Tordsson (9) highlights that friluftsliv arises from specific historical, social, and cultural conditions. From a philosophical perspective, it can be argued that friluftsliv has allowed young people to engage more closely with social and existential issues, and during the pandemic, they have used the countryside as an escape from one reality into another. Indeed, perhaps pursuing friluftsliv was the young people's response to “…nature's open appeal” (20). It could also have been crucial for young people to utilize friluftsliv as a means to experience life, gain self-affirmation, and identify with something, or someone, through their friluftsliv (14).

### Strengths and limitations of the study

This study possesses several strengths. One major advantage was that all of the 287 students who were randomly selected answered the questionnaire. We contend that the sample likely presents a representative sample of Norwegian youth. This assertion is supported by the fact that the gender distribution in our study mirrors the actual gender distribution in Norwegian schools. The results were recorded from the youths’ own answers, using a five-point Likert scale with questions possessing strong face validity. Five-point Likert scales are commonly used in questionnaire studies, and the five answer options—two at each end and one in the middle, and the option between the middle and each end—are both valid and reliable. Data quality, internal consistency, and discriminative validity suggest that the five-point scale version should be used in future related research (40). Moreover, to the best of the authors’ knowledge, this constitutes the first study to be undertaken in Norway regarding this specific research area. Furthermore, collecting data for a cross-sectional study at such a difficult time for individuals and families related to physical activity and social life is of great importance. The fact that both statistical analyses and qualitative analyses (to a certain degree) were used is also a strength. Nevertheless, our study also has certain limitations. In general, the use of a self-reported questionnaire introduces certain constraints in terms of validity and reliability. In addition, some of the research questions were not validated in other studies, but were developed by some of the researchers of the current study. However, we emphasise that all of the questions used in this study possess high face validity (41) because the 12 friluftsliv activities are defined and well-known among Norwegians. Accurately recalling the number of trips of each activity, however, could pose a challenge and might compromise reliability. This is especially evident in the data collected about pre-pandemic friluftsliv activities, which were gathered using a retrospective procedure. Although this study included data from an open-ended question, which is a strength, these data have certain limitations in contributing to a profound understanding of the phenomenon. This is primarily due to the use of a single question, which often elicited little written reflections by the participants.

## Conclusion

This study demonstrates that the activity level of friluftsliv activities declined significantly by 12% during the pandemic. Despite the decrease in both traditional and modern friluftsliv activities, the decline was much larger in the modern activities. Another major finding was that more respondents emphasized the significance of friluftsliv activities in maintaining social relationships, in general, and relationships with friends, family, and girlfriend/boyfriends, during the pandemic than before the pandemic. Our study also indicates that, even though friluftsliv activity decreased during the pandemic, young people found these activities to be particularly vital for creating a sense of community during the pandemic. Our findings highlight friluftsliv activities as a crucial arena for young people to experience social belonging and physical activity during pandemic situations. While other activities are not permitted or curtailed in such situations, friluftsliv activities are allowed due to their nature of being conducted in the fresh air. Our results indicate that increased attention should be given to friluftsliv activities in pandemic situations. Such a strategy will not only satisfy the psychological need for relatedness, but also promote physical health and mental well-being.

## Data Availability

The raw data supporting the conclusions of this article will be made available by the authors, without undue reservation.
